# Intraoperative transcystic laparoscopic common bile duct stone clearance with SpyGlass™ discover during emergency and elective cholecystectomy: a single-center case series

**DOI:** 10.1186/s13017-023-00529-0

**Published:** 2024-03-04

**Authors:** Paola Fugazzola, Carlo Maria Bianchi, Francesca Calabretto, Enrico Cicuttin, Francesca Dal Mas, Tommaso Dominioni, Marcello Maestri, Aurelio Mauro, Alice Podestà, Matteo Tomasoni, Francesco Brucchi, Jacopo Viganò, Luca Ansaloni, Andrea Anderloni, Lorenzo Cobianchi

**Affiliations:** 1 Division of General Surgery, IRCCS Policlinico San Matteo Foundation, via Camillo Golgi 19, 27100 Pavia, PV Italy; 2 Department of Clinical, Diagnostic and Pediatric Sciences, University of Pavia, via Alessandro Brambilla, 74, 27100 Pavia, PV Italy; 3 Breast Surgery Unit, San Raffaele Hospital of Milan, Milan, Italy; 4 Department of Management, Ca’ Foscari University of Venice, Venice, Italy; 5 Endoscopy Unit, Fondazione IRCCS Policlinico San Matteo, Pavia, Italy; 6 University of Milano, Milan, Italy; 7 Collegium Medicum, University of Social Sciences, Łodz, Poland

**Keywords:** Case series, Choledochoscopy, Choledocholithiasis, Cholangioscopy, SpyGlass

## Abstract

**Background and study aim:**

The development of a new cholangioscope, the SpyGlass™ Discover (Boston Scientific), has allowed the laparoscopic transcystic common bile duct exploration and stone clearance. The possibility of simultaneous treatment of choledocholithiasis during early laparoscopic cholecystectomy offers the opportunity to enormously reduce the time between acute cholecystitis diagnosis and the execution of cholecystectomy with better outcomes for patients. Furthermore, an altered anatomy of the gastrointestinal tract is not an obstacle to this technique. The aim of the study was to determine whether this new procedure is feasible, safe, and effective.

**Patients and methods:**

The investigation employs a retrospective case series study including all consecutive patients with a diagnosis of common bile duct stones undergoing cholecystectomy and intraoperative laparoscopic common bile duct clearance using SpyGlass™ Discover at IRCCS Policlinico San Matteo in Pavia (Italy). Eighteen patients were included from May 2022 to May 2023.

**Results:**

A complete clearance of the common bile duct was obtained in 88.9% of patients. The mean postoperative length of stay was 3 days. No major complications occurred. After a median follow-up of 8 months, no recurrence of biliary events or readmissions occurred.

**Conclusion:**

This procedure has proven to be feasible, safe, and effective.

**Supplementary Information:**

The online version contains supplementary material available at 10.1186/s13017-023-00529-0.

## Introduction

Common bile duct stones (CBDS) are present in 10–20% of patients with gallstones and in 5–15% of patients with acute calculous cholecystitis (ACC) [1].

In order to diagnose and treat biliary obstruction, endoscopic retrograde cholangiopancreatography (ERCP) stands as the gold standard [2]. However, ERCP is rarely used for diagnostic purposes, while second level examination, i.e., endoscopic ultrasound (EUS) or magnetic resonance cholangiopancreatography (MRCP), is often needed before ERCP for the diagnosis of CBDS. However, ERCP, EUS, and MRCP are costly and might not be readily available. The latter may cause ACC patients' surgical therapy to be delayed and decrease a favorable outcome [3–6]. Indeed, it is widely recognized and stated by international guidelines [1] that the best treatment for patients with ACC is early laparoscopic cholecystectomy (ELC). The definition of ELC is cholecystectomy performed as soon as possible from the moment of ACC diagnosis, preferably within 72 h following admission [3–6]. Among the reasons that make it challenging to achieve this aim (i.e., performing laparoscopic cholecystectomy as soon as possible) is the diagnosis of the presence of CBDS and their treatment by ERCP before performing laparoscopic cholecystectomy.

Recent pieces of research reported how simultaneous laparoscopic cholecystectomy and intraoperative ERCP stand as safer procedures for individuals with cholecystocholedocholithiasis. Such a joint procedure might facilitate intubation, decrease the need for additional stone removal operations, reduce hospital stays, and lessen postoperative problems, such as pancreatitis and stone residue [7]. However, in some cases, an altered anatomy of the gastrointestinal tract (e.g., after gastric, pancreatic, or biliary surgery) or a particular conformation of the duodenal papilla may prevent the papilla from being reached or intubated, making it impossible to perform ERCP. Furthermore, performing an intraoperative ERCP during cholecystectomy, especially in an emergency setting, is often burdened by major organizational and coordination issues among different specialists and the operating room.

In recent years, the development of a new cholangioscope, the SpyGlass™ Discover (Boston Scientific) [8–12] with an outer diameter of 3.5 mm, has allowed the laparoscopic transcystic CBD exploration and CBDS clearance. The possibility of simultaneous treatment of CBDS with SpyGlass™ Discover, after intraoperative cholangiography (IOC), during ELC offers the opportunity to enormously reduce the time between ACC diagnosis and the execution of ELC with better outcomes for these patients. Furthermore, an altered anatomy of the gastrointestinal tract is not an obstacle to this technique. The article presents a single-center case series including patients undergoing intraoperative laparoscopic transcystic treatment of CBDS using SpyGlass™ Discover during cholecystectomy.

## Material and methods

### Design

The present investigation stands as a retrospective case series study including all consecutive patients with a diagnosis of CBDS undergoing cholecystectomy and intraoperative laparoscopic CBD clearance using the cholangioscope SpyGlass™ Discover at IRCCS Policlinico San Matteo in Pavia (Italy). On a one-year horizon, from May 2022 to May 2023, 18 patients were included. The aim of the study was to determine whether this new procedure is feasible, safe, and effective.

### Ethical considerations

Patients provided written informed consent. The study was carried out in line with the Helsinki Declaration. The ethics approval was not required for the collection, analysis, and publication of the retrospectively obtained and anonymized data for this case series.

### Studied variables

Preoperative, intraoperative, and postoperative data were collected for all patients. Postoperative complications were defined with Clavien–Dindo grade. All patients were contacted by phone for follow-up.

## Results

From May 2022 to May 2023, 18 patients with a diagnosis of CBDS underwent cholecystectomy and intraoperative laparoscopic CBD clearance using SpyGlass™ Discover at IRCCS Policlinico San Matteo in Pavia (Italy). The details of the patients and their clinical outcomes are reported in Table 1.

**Table 1:** Characteristics and outcomes of patients undergoing common bile duct exploration and clearance with SpyGlass Discover

No.	Age	Admission	Reason for admission	Preoperative attempt to ERCP	Preoperative imaging	Previous abdominal surgery	ASA	POSSUM-PS	Operative time (min)	Failure of cystic duct intubation	Complete clearance of CBD	ICU admission	Postoperative LOS	Total LOS	Postoperative complications	Clavien–Dindo grade	Need of postop ERCP
1	73	Emergency	ACC with suspected CBDS	No	EUS	Yes	2	20	240	0	1	0	3	11	0	0	0
2	75	Emergency	ACC with suspected CBDS	No	CT	Yes	2	21	300	0	1	0	3	15	0	0	0
3	71	Emergency	ACC with suspected CBDS	No	US	No	2	22	180	0	1	0	2	10	0	0	0
4	70	Emergency	ACC with suspected CBDS	No	US	Yes	2	32	180	0	1	0	5	9	Biliary leak from drainage, spontaneously resolved	1	0
5	51	Emergency	AP with suspected CBDS	No	CT	Yes	2	19	150	0	1	0	2	8	0	0	0
6	80	Emergency	ACC with suspected CBDS	No	US	Yes	2	24	240	0	1	0	2	5	0	0	0
7	85	Emergency	ACC with suspected CBDS	No	MRCP	Yes	2	17	180	0	1	0	4	8	Asymptomatic rise of serum lipases, spontaneously resolved	1	0
8	69	Emergency	ACC with suspected CBDS	No	CT	Yes	2	17	150	0	1	0	5	6	0	0	0
9	82	Elective	CBDS	No	MRCP	Yes	2	23	120	0	1	0	1	3	0	0	0
10	54	Emergency	ACC with suspected CBDS	No	US	No	2	15	210	0	1	0	8	15	0	0	0
11	65	Emergency	ACC and AP with suspected CBDS	No	US	No	2	16	150	0	1	0	1	13	0	0	0
12	84	Emergency	ACC with suspected CBDS	No	US	Yes	2	36	210	1	0	0	3	12	0	0	1
13	76	Elective	CBDS	No	US	Yes	2	18	150	0	1	0	2	5	0	0	0
14	85	Emergency	ACC and AP with CBDS	No	TC	Yes	2	19	210	0	1	0	2	8	0	0	0
15	79	Elective	AP with suspected CBDS	Yes, failure of major papilla intubation	MRCP	No	2	15	120	0	1	0	2	5	0	0	0
16	83	Emergency	AP with suspected CBDS	Yes, failure of major papilla intubation	US	Yes	2	26	210	0	1	0	2	15	0	0	0
17	76	Emergency	ACC with suspected CBDS	No	CT	No	2	28	140	0	1	1 (for associated comorbidity)	8	9	0	0	0
18	65	Elective	CBDS	No	US	Yes	2	14	210	0	0	0	3	3	Biliary leak from drainage, spontaneously resolved	1	1

ACC, acute calculous cholecystitis; AP, acute pancreatitis; CBDS, common bile duct stones; EUS, endoscopic ultrasonography; CT, computed tomography; MRCP, magnetic resonance cholangiopancreatography; CBD, common bile duct

The mean age of patients was 73.5 ± 10.1, and their mean POSSUM physiological score was 21.2. 77.8% of patients were admitted to the ward from the Emergency Department. 66.7% had a diagnosis of ACC, and the 27.8% of acute biliary pancreatitis. Four patients were admitted for an elective intervention for a diagnosis of CBDS and impossibility to perform ERCP. Three of them had a previous esophageal-gastric surgery, while one had an earlier failure of major papilla intubation. The diagnosis of CBDS was made with ultrasound (US) in 44.4% of patients, with CT scan in 27.8% and with EUS or MRCP in 22.2%, as reported in Table 2. In two patients (11.1%), an ERCP was attempted and failed for the impossibility of papilla intubation. In our series, the size of the main biliary duct stones (obtained from preoperative imaging) ranged from sludge to 1 cm.

**Table 2:** Preoperative patients characteristics

Characteristics		Mean ± SD Median (IQR) N (%)
Age (years)		73.5 ± 10.1 75.5 (69.0–82.0)
Sex	Male	11 (61.1)
	Female	7 (38.9)
Hospital admission	Emergency	14 (77.8)
	Elective	4 (22.2)
Patients with ACC		12 (66.7)
Patients with AP		5 (27.8)
Imaging for CBDS diagnosis	Only US	8 (44.4)
	CT scan	5 (27.8)
	MRCP	3 (16.7)
	EUS	1 (5.6)
POSSUM-PS		21.2 ± 6.1 19.5 (17.0–24.0)
PCR (mg/dl)		7.1 ± 10.4 2.4 (0.9–8.3)
PCT (ng/mL)		1.8 ± 1.7 1.3 (0.3–3.2)
Serum lipase (U/L)		66.3 ± 62.9 36.0 (30.5–71.5)
Gamma-GT (mU/mL)		137.8 ± 148.8 61.0 (29.5–217.0)
AP (UI/L)		125.4 ± 48.6 114.0 (85.5–165.0)
Direct bilirubin (mg/dl)		2.5 ± 2.1 0.9 (0.9–3.9)

ACC, acute calculous cholecystitis; AP, acute pancreatitis; CBDS, common bile duct stones; US, ultrasonography; EUS, endoscopic ultrasonography; CT, computed tomography; MRCP, magnetic resonance cholangiopancreatography; POSSUM-PS, POSSUM physiological score; PCR, protein-c reactive; PCT, procalcitonin; gamma-GT, gamma-glutamyl transferase; AP, alkaline phosphatase

**Fig. 1:**
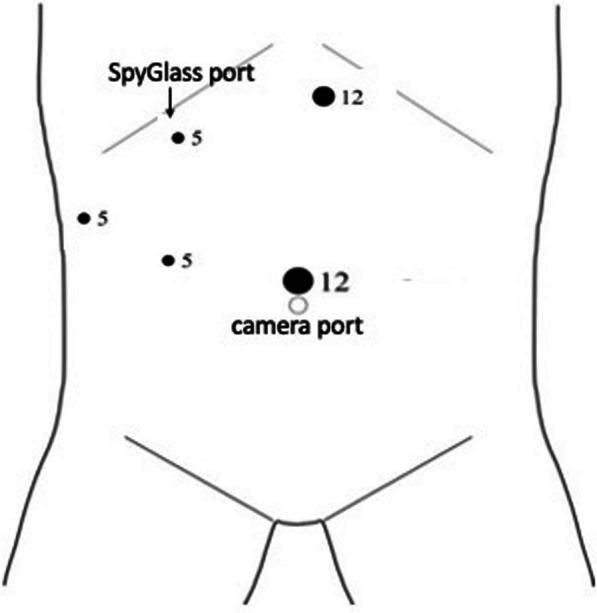
Trocar position

Laparoscopic cholecystectomy was performed in the American position. Laparoscopic access was performed by an optical 12mm umbilical trocar after Verres needle insufflation in Palmer point. In patients with previous surgeries, we entered the abdomen with an open technique and placed a 12-mm trocar at the umbilicus to insufflate the abdomen. Then, three more trocars were placed: an epigastric 12 mm and two 5-mm trocars in right flank (one on the right anterior axillary line and one in the midclavicular line), as highlighted in the following Fig. 1.

**Fig. 2:**
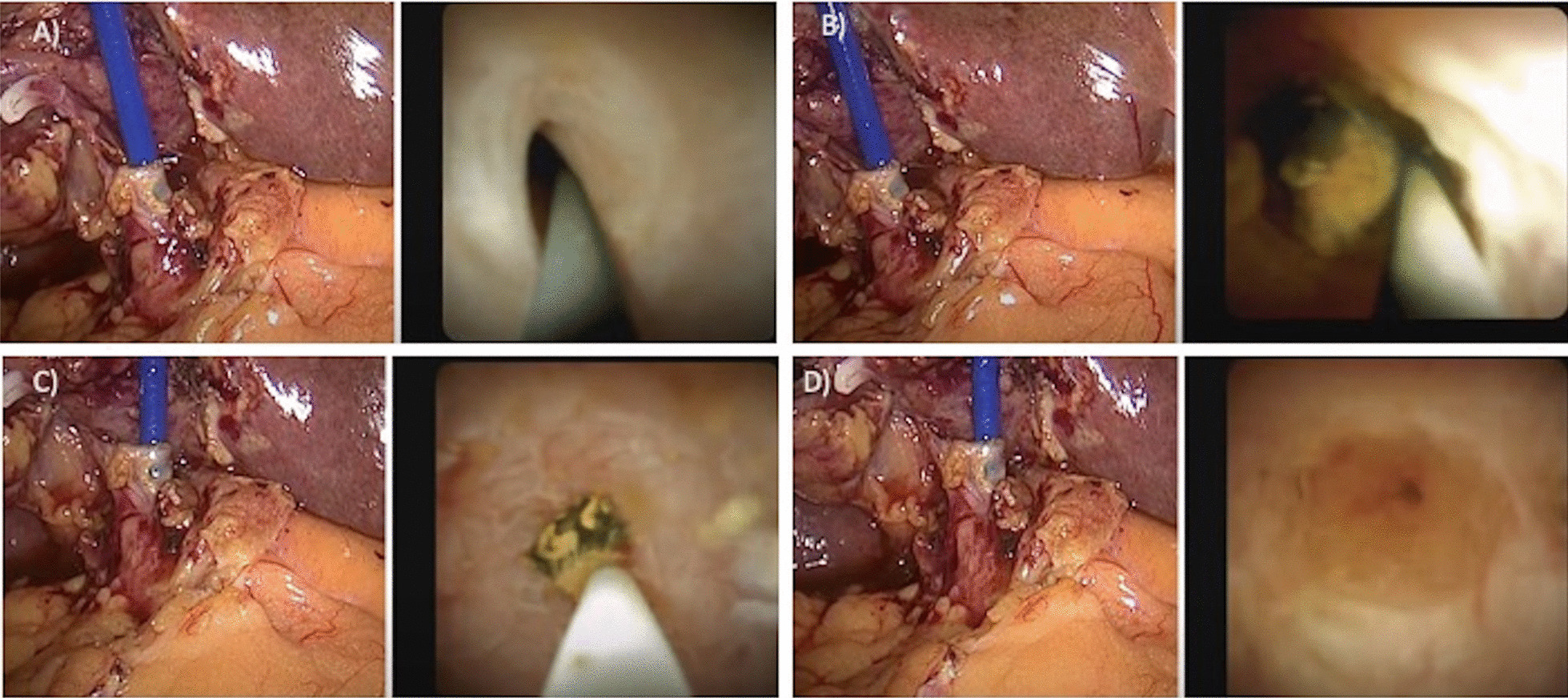
Laparoscopic cholangioscopy and common bile duct stones removal using SpyGlass™ Discover: **A** intubation of the cystic duct with the cholangioscope after positioning of guide wire; **B** cholangioscopy and common bile duct stones finding; **C** removal of stones, which are pushed beyond the duodenal papilla; **D** complete clearance of the common bile duct

The cystic duct was isolated, and a transcystic intraoperative cholangiography (IOC) was performed to confirm the presence of CBDS. In case of CBDS confirmation, a fifth 5-mm trocar in the right hypochondrium was placed for cholangioscope SpyGlass™ Discover insertion. The cystic duct was intubated with the cholangioscope after positioning of the guide wire, and a cholangioscopy of the CBD was performed (Fig. 2A, B, Additional file 1: Video 1). In cases of very small or winding cystic duct, a complete cystic duct isolation and intubation of the duct more proximal to the CBD was necessary. The major papilla was passed, and the SpyGlass™ gently pushed up into the duodenum. Then, the SpyGlass™ was retracted in the CBD, and the stones removed with one of the following techniques, depending on the characteristics of the stones and the papilla:

- In the case of sludge, it was pushed into duodenum through the papilla with pressure washing using the washing channel of the instrument;
- In the case of small stones (smaller than cystic duct diameter), they were removed through the cystic duct using the basket (Additional file 1: Video 1);
- In the case of big stones and of a permissive papilla, stones were pushed into the duodenum under direct vision through the papilla using the cholangioscope (Fig. 2C, D).

After CBDS removal, a transcystic IOC was performed to confirm complete CBD clearance. The cystic duct was closed with Hemolock clip or with laparoscopic suture with PDS wire and the cholecystectomy completed. A drain was positioned under the liver and removed, if no biliary leak occurred, in second postoperative day.

We obtained a complete clearance of the CBD using the cholangioscope SpyGlass™ Discover in the 88.9% of patients. Two patients (11.1%) needed postoperative ERCP.

In one patient, we failed to intubate the cystic duct due to its small diameter, and a postoperative ERCP for CBDS removal was necessary.

In one patient with seven quite big stones in the CBD and a fibrotic papilla, we failed to pass it and reach the duodenum. Then, because we could not make the stones overcome the papilla, we removed five stones through the cystic duct using the basket. However, one stone was too large to pass the cystic duct, and we failed to reach a complete CBD clearance. This patient had a low-flow biliary leak from drainage on the first postoperative day, which spontaneously resolved. The patient underwent ERCP for residual CBDS removal. During the procedure, a transpapillary cholangiography was performed without biliary leak evidence.

The mean operative time was 186 min. No intraoperative complications occurred.

After the first procedure that was performed in collaboration with endoscopists, all the following procedures were performed by a team of general surgeons only. The mean postoperative LOS was 3 days and the total mean LOS was 9 days. Three patients (16.6%) had minor postoperative complications (Clavien–Dindo 1): two patients had a low-flow biliary leak from drainage and one patient had an asymptomatic rise of serum lipase, all spontaneously resolved in 1–2 days. No major complications occurred (Table 3). After a median follow-up of 8 months (IQR 149–356 days), no new biliary events, post-discharge complications, or readmissions occurred.

**Table 3:** Intraoperative and postoperative outcomes

Characteristics	Mean ± SD Median (IQR) *N* (%)
Operative time (minutes)	186.1 ± 47.2 180.0 (150.0–210.0)
Intraoperative complication	0 (0.0)
Failure of cystic duct intubation	1 (5.6)
Complete clearance of CBD	16 (88.9)
Postoperative LOS (days)	3.2 ± 2.1 2.5 (2.0–4.0)
Total LOS (days)	8.9 ± 4.0 8.5 (5.0–12.0)
Postoperative minor complications (CD < 3)	3 (16.7)
Postoperative major complications (CD ≥ 3)	0 (0.0)
Need of postoperative ERCP	2 (11.1)

LOS, length of stay; CBD, common bile duct; CD, Clavien–Dindo

## Discussion

The SpyGlass™ Digital Catheter is intended to provide direct visualization and guide optical and accessory devices for diagnostic and therapeutic applications during endoscopic procedures in the pancreaticobiliary system. It is specifically designed for the complete visualization of the extrahepatic biliary tree. The SpyGlass™ incorporates a perfusion channel to retain a good field of view and a greater optical resolution than traditional bile duct scopes. Additionally, it has a four-way angle which allows to easily visualize narrow and intricate ducts, such as the cystic and intrahepatic bile ducts [9]. The characteristics of SpyGlass™ Discover allows the percutaneous or laparoscopic transcystic exploration of CBD and the CBDS removal. Furthermore, SpyGlass™-guided electrohydraulic or laser lithotripsy can be used for difficult common bile duct stones not amenable to conventional endoscopic therapy.

Recently, there have been several reports regarding the peroral use of the SpyGlass™ DS for the diagnosis of indeterminate biliary stricture [8] and cystic duct neoplasm [9]. Furthermore, the use of SpyGlass™ technology for single-operator transpapillary [10] or percutaneous [11] cholangioscopy for removing difficult stones has been described too.

To our knowledge, this case series is the second and the largest published in the literature that includes patients undergoing intraoperative laparoscopic transcystic treatment of CBDS using SpyGlass™ Discover during cholecystectomy.

Up to now, only a series of four cases of patients undergoing intraoperative laparoscopic transcystic treatment of CBDS using SpyGlass™ Discover during cholecystectomy was reported by Kouli et al. with good outcomes [12].

Also a case of a patient with a huge stone in the CBD successfully treated with laparoscopic choledochotomy and cholangioscopy using SpyGlass™ Discover was reported by Palermo et al. [13]. However, probably for the huge diameter of the stone, the CBD exploration in this case was not performed with the transcystic technique.

There are many advantages of laparoscopic CBDS removal using SpyGlass™ Discover during cholecystectomy. First of all, the laparoscopic cholangioscopy is likely burdened with low risks due to the fact that it is not necessary to perform papillosphincterotomy. Our series confirms this low-risk profile, in particular, it showed no major complication after the procedure, no acute pancreatitis or post-procedural bleeding. Furthermore, this procedure is readily available, because the operation could be entirely performed by acute care surgeons or general surgeons during laparoscopic cholecystectomy, without needing endoscopic training. In patients with ACC with suspected CBDS, the opportunity to simultaneous treatment of CBDS during ELC, offers the possibility of enormously reducing the time between ACC diagnosis and the execution of ELC with better outcomes for these patients [3–6].

It should be noted that in our series, the total LOS is quite long, as, until recently, in our institution, the device was available only under special request due to the previous hospital’s administrative policy. Because the device is currently fully functional, we expect the preoperative time to be shorter. Indeed, most patients with ACC and associated CBDS are likely to receive ELC and simultaneous CBDS removal within 72h from admission.

Another advantage of this technique is the possibility to explore the CBD also in patients with altered gastrointestinal anatomy or in case of difficult access to the papilla (e.g., in the case of intradiverticular papilla). In our series, two patients with CBDS underwent successful laparoscopic CBDS removal after a failed ERCP due to the difficulty of papilla intubation.

The present series shows the safety and effectiveness of the intraoperative laparoscopic transcystic treatment of CBDS using SpyGlass™ Discover during cholecystectomy, reporting a success rate of 89% and no major complications.

The present study has evident limitations. First of all, this is a retrospective case series. The sample size is limited, and lacks a control group. However, this series represents one of the first pieces of evidence of the innovative application of SpyGlass™ Discover cholangioscope. Results are encouraging, and it emerges how the procedure is feasible, effective, and safe. The next step will be to compare the efficacy and risk profile of this procedure with ERCP followed by cholecystectomy. In this regard, the "INtraoperative Approach with eventual Clearance of Common bilE duct by SpyGlass™ Discover vs Sequential strategy in patients with acute calculus cholecystitis and Intermediate/high risk of common BiLE duct stone (INACCESSIBLE)" will soon begin in Italy. In this trial, patients with ACC with high/intermediate risk of CBDS, according to the score by Khoury et al. [14, 15], will be randomized to receive ERCP + ELC in two-stage procedures (control group) or simultaneous ELC + SpyGlass™ cholangioscopy and CBDS removal (study group).

### Supplementary Information

**Additional file 1: Video 1**. Laparoscopic cholangioscopy and common bile duct stones removal using SpyGlass™ Discover.

## Data Availability

The datasets generated and/or analyzed during the current study are not publicly available but are available from the corresponding author at reasonable request.
